# Neat Way of Giving Quinine

**Published:** 1890-07

**Authors:** 


According to Dr. L. G. Doane, of New York City, a neat way of giving quinine is as follows :

℞—Quinine . . . . . . . . . . . . . . . . . . . . . . . . 1 grain

Chocolate . . . . . . . . . . . . . . . . . . . . . . . . 1 scruple

Children, he says, will take quinine in this form and cry for more.

---

Solution of Chloral Hydrate, grains five to the ounce of water, will clear the hair of dandruff and prevent it falling out from this cause.—*American Lancet.*

